# Clinical Predictors Influencing the Length of Stay in Emergency Department Patients Presenting with Acute Heart Failure

**DOI:** 10.3390/medicina56090434

**Published:** 2020-08-27

**Authors:** Pungkava Sricharoen, Phichayut Phinyo, Jayanton Patumanond, Dilok Piyayotai, Yuwares Sittichanbuncha, Chaiyaporn Yuksen, Khanchit Likittanasombat, Ekpaiboon Cheuathonghua

**Affiliations:** 1 Department of Emergency Medicine, Faculty of Medicine, Ramathibodi Hospital, Mahidol University, Bangkok 10400, Thailand; pungkawa@hotmail.com (P.S.); yuwares.sit@mahidol.ac.th (Y.S.); chaipool0634@hotmail.com (C.Y.); ch.ekpaiboon@gmail.com (E.C.); 2 Department of Family Medicine, Faculty of Medicine, Chiang Mai University, Chiang Mai 50200, Thailand; 3 Center for Clinical Epidemiology and Clinical Statistics, Faculty of Medicine, Chiang Mai University, Chiang Mai 50200, Thailand; jpatumanond@gmail.com; 4 Department of Medicine, Faculty of Medicine, Thammasat University, Pathum Thani 12121, Thailand; dilokpiyayotai@yahoo.com; 5 Department of Internal Medicine, Faculty of Medicine, Ramathibodi Hospital, Mahidol University, Bangkok 10400, Thailand; khanchit.lik@mahidol.ac.th

**Keywords:** heart failure, emergency department, predictors, risk stratification, length of stay

## Abstract

*Background and objectives:* Acute heart failure is a common problem encountered in the emergency department (ED). More than 80% of the patients with the condition subsequently require lengthy and repeated hospitalization. In a setting with limited in-patient capacity, the patient flow is often obstructed. Appropriate disposition decisions must be made by emergency physicians to deliver effective care and alleviate ED overcrowding. This study aimed to explore clinical predictors influencing the length of stay (LOS) in patients with acute heart failure who present to the ED. *Materials and Methods:* We conducted prognostic factor research with a retrospective cohort design. Medical records of patients with acute heart failure who presented to the ED of Ramathibodi Hospital from January to December 2015 were assessed for eligibility. Thirteen potential clinical predictors were selected as candidates for statistical modeling based on previous reports. Multivariable Poisson regression was used to estimate the difference in LOS between patients with and without potential predictors. *Results:* A total of 207 patients were included in the analysis. Most patients were male with a mean age of 74.2 ± 12.5 years. The median LOS was 54.6 h (Interquartile range 17.5, 149.3 h). From the multivariable analysis, four clinical characteristics were identified as independent predictors with an increase in LOS. These were patients with New York Heart Association (NYHA) functional class III/IV (+72.9 h, 95%Confidence interval (CI) 23.9, 121.8, *p* = 0.004), respiratory rate >24 per minute (+80.7 h, 95%CI 28.0, 133.3, *p* = 0.003), hemoglobin level <10 mg/dL (+60.4 h, 95%CI 8.6, 112.3, *p* = 0.022), and serum albumin <3.5 g/dL (+52.8 h, 95%CI 3.6, 102.0, *p* = 0.035). *Conclusions:* Poor NYHA functional class, tachypnea, anemia, and hypoalbuminemia are significant clinical predictors of patients with acute heart failure who required longer LOS.

## 1. Introduction

Heart failure is a malignant cardiac syndrome with high morbidity and mortality rate. It is considered a major public health burden that affects around 26 million people worldwide [1]. The reported prevalence of heart failure in Southeast Asian countries, including Thailand, was higher than in other regions of the world [2], ranging from 5 to 7% [3]. Even though heart failure is generally described as a chronic debilitating disease, most patients ultimately experience acute symptoms that trigger emergency department (ED) visits [4]. In the United States, more than 80% of patients who visited the ED with acute heart failure symptoms were finally admitted to the hospital [5]. These patients usually require lengthy and repeated hospital admission, which results in an immense economic burden [6].

According to Ramathibodi Hospital statistics in 2016, 583 patients with acute heart failure visited the ED during the one-year period. Of these numbers, only 61 (10.4%) were hospitalized, which was much lower than the admission rate of the United States [5]. This was due to the limited number of in-patient beds available, which leads to ED overcrowding. As high as 239 (42.0%) patients with acute heart failure were retained in the ED for more than 24 h with a maximum length of stay (LOS) of 9 days. Although a large proportion of patients with acute heart failure require hospitalization, the remaining low-risk patients with mild symptoms could be managed in the ED and could safely be discharged for home [4]. For this reason, emergency physicians must make a proper and effective decision, whether to admit the patients to in-patient wards, to admit the patients to the observational unit, or to discharge the patients from the ED [7]. An inappropriate decision could lead to improper patient disposition, which would further aggravate the ED throughput. The use of prognostic guidance or the knowledge of factors that influence the patient’s LOS might assist emergency physicians in their clinical decision making.

Several studies have addressed predictors associated with longer LOS in patients with acute heart failure, such as elderly patients, patients with multiple comorbidities (e.g., chronic kidney disease, ischemic heart disease, and diabetes), patients with poor functional class, patients with anemia, hyponatremia, and hypoalbuminemia [8,9,10,11,12]. However, most studies were performed in developed countries where patients’ clinical and health care characteristics significantly differed from Thailand [13]. The generalization of results from those studies to our setting might be inappropriate. Only one study from Thailand had examined factors that influence the duration of hospitalization in patients with heart failure [14]. However, only hospitalized patients were included in this study, as in most previous studies. This study intended to explore the clinical predictors associated with an increase in total LOS of patients with acute heart failure who presented to the ED of Ramathibodi Hospital as a representative of university-affiliated hospitals in the center of Bangkok, Thailand.

## 2. Materials and Methods

We conducted prognostic factor research using a retrospective cohort design. All patient data were collected through the electronic medical record database of Ramathibodi Hospital from January 2016 to December 2017. Ramathibodi Hospital is a university hospital of the Faculty of Medicine Ramathibodi Hospital, Mahidol University, a super tertiary care center with 1300 in-patient beds. In 2016, about 167 patients visited the ED each day, with an admission rate of 13.1%. Around 27.6% of the patients stayed in the ED for more than 24 h.

The ethical consideration of the study protocol was granted by The Committee on Human Rights Related to Research Involving Human Subjects, Faculty of Medicine Ramathibodi Hospital, Mahidol University [MURA2017/650] and The Human Research Ethics Committee of Thammasat University [COA070/2561]. Informed consent was not required as all patient data were retrospectively collected and were anonymous.

All adult patients (aged ≥ 18 years) records with the final diagnosis based on the 10th revision of the International Statistical Classification of Diseases and Related Health Problems (ICD-10) of congestive heart failure (I500), hypertensive heart disease with congestive heart failure (I110 and I130), or fluid overload (E877) were screened for eligibility. The inclusion criteria were adult patients who presented to the ED with an acute dyspneic episode with other clinical symptoms suggestive of acute heart failure, fulfilled the Framingham heart failure diagnostic criteria [15], and had an N-terminal pro b-type natriuretic peptide level (NT-ProBNP) > 300 pg/mL [16,17,18]. Our study’s criteria for the diagnosis of acute heart failure was according to the 2016 ESC guidelines for the diagnosis and treatment of acute and chronic heart failure [19], which recommends the use of NT-proBNP in the differentiation of acute heart failure from non-cardiogenic causes of dyspnea. Patients who died during admission, who were referred to or from other hospitals, were discharged against advice, were miscoded as acute heart failure, and had incomplete data on predictors or outcomes were excluded. In patients who revisited the hospital within 14 days, their latest records were used.

Patient demographic and clinical characteristics including age, gender, ethnicity, underlying conditions (i.e., chronic kidney disease, ischemic heart disease, diabetes mellitus, atrial fibrillation), mode of presentation (i.e., functional class according to the New York Heart Association (NYHA)), initial vital signs and hemodynamic parameters (i.e., systolic blood pressure, heart rate, respiratory rate, and oxygen saturation), and initial laboratory investigation (i.e., ProBNP, serum sodium, hemoglobin, and serum albumin) were collected. The presence of any comorbidity was based on previous documentation of the disease diagnosis that appeared in medical records and ICD-10 codes at discharge diagnosis. In this study, chronic kidney disease was defined as a glomerular filtration rate of <60 mL/min/1.73 m^2^. If transthoracic echocardiograms were performed during admission, the data on ejection fraction was collected. Data on the LOS were defined as the time from the patient visit to the ED to the time that the patient was discharged from the hospital, collected in the unit of hours.

We explored thirteen candidate predictors for their contributions to the total LOS. All pre-selected risk factors and their cutoffs were chosen based on previous studies which addressed their association with prolonged LOS or mortality: age > 65 years [20], male gender [21], chronic kidney disease [10], ischemic heart disease [10], diabetes mellitus [10], atrial fibrillation [22], NYHA functional class III/IV [8], systolic blood pressure < 100 or > 140 [23], respiratory rate > 24 per minute [24], ProBNP ≥ 1800 pg/mL [17], serum sodium < 135 mmol/L [25], hemoglobin < 10 g/dL [26], and serum albumin < 3.5 g/dL [21].

Continuous variables were presented with mean ± standard deviation or median with interquartile range, in appropriation with data distribution. Categorical variables were presented with frequency and percentage within each category. To explore the effect of each predictor on the LOS, univariable and multivariable Prior to statistical modeling, variance inflation factor (VIF) was used for diagnosis of collinearity among predictor variables. Poisson regression for count data was used to estimate the difference in the LOS, contrasting between patients with and without the candidate predictors. As the objective of our study was exploratory, independent predictors of LOS were defined as statistically significant predictors in the full multivariable model. Sensitivity analysis was performed by backward elimination of non-significant predictors, and by including serum creatinine value during statistical modeling to account for possible interaction between acute heart failure and acute kidney injury [27]. A two-sided *p* < 0.05 was pre-set for statistical significance. All statistical analyses were performed by Stata 16 (StataCorp, College Station, TX, USA).

## 3. Results

**Figure 1:**
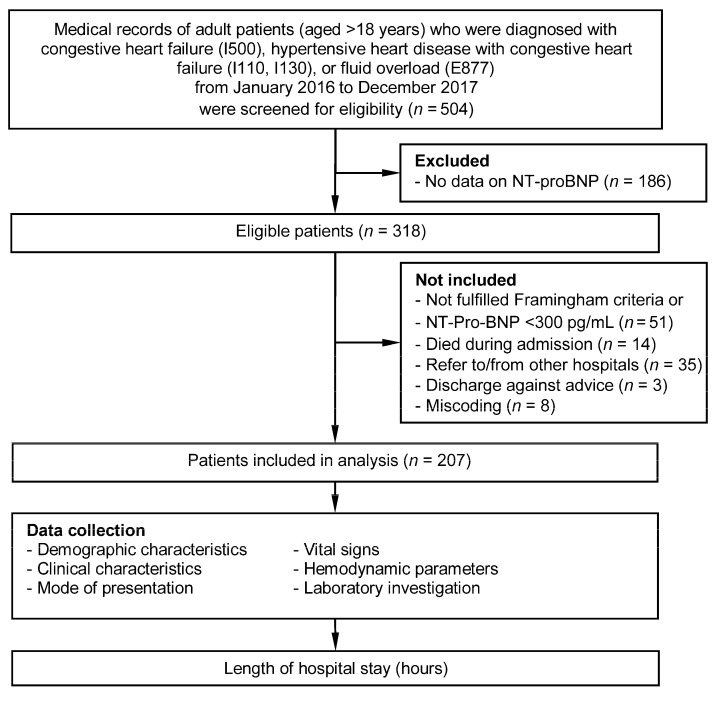
Data flow diagram.

During the study period, a total of 504 adult patients visited the ED with symptoms suggestive of acute heart failure. NT-proBNP values were missing in 186 patients and were excluded from the analysis to ensure the accuracy of the heart failure diagnosis. Of the remaining 318 patients, 51 did not fulfill the Framingham diagnostic criteria or had NT-proBNP value < 300 pg/mL, 35 were referred to or from other hospitals, 14 died during admission, 3 were discharged against advice, and 8 were miscoded with acute heart failure. The remaining 207 patients were included in the analysis (Figure 1). More than half of the patients were male (62%) with a mean age of 74.2 ± 12.5 years. Only 23 (11.1%) were without any documented comorbidity. Table 1 describes the remaining details of the relevant clinical characteristics of the study patients. The comparison of baseline clinical characteristics between patients who had data on NT-proBNP and patients who did not have is shown in Supplementary Table S1. The data on ejection fraction (EF) from transthoracic echocardiography were available in 94.2% of patients. There were 102 (52.3%) patients with heart failure with preserved ejection fraction (HFpEF, EF ≥ 50%), 48 (24.6%) heart failure with with mid-range ejection fraction (HFmrEF, EF 40–49%), and 45 (23.1%) with heart failure with reduced ejection fraction (HFrEF, EF < 40%) according to the European Society of Cardiology guideline [19]. Thirteen (6.3%) patients were admitted to in-patient wards, 59 (28.5%) patients were admitted to an intensive care unit, and 135 (65.2%) were treated in the ED and discharged home after clinical improvement.

The median total LOS was 54.6 h, with an interquartile range of 17.5 and 149.3 h. The minimum and maximum LOS were 1.7 and 1322.8 h. The estimated Poisson means of LOS between patients with or without potential predictors appeared in Table 2. From the univariable analysis, all pre-specified prognostic factors other than the male gender showed a statistically significant difference in LOS (Table 2). In addition, the presence of all predictors significantly increased the LOS, except for systolic blood pressure < 100 or >140 mmHg. In multivariable analysis, only four predictors were identified as independent factors associated with increasing LOS. These were NYHA functional class III/IV (+72.9 h, 95%CI 23.9, 121.8, *p* = 0.004), respiratory rate > 24 per minute (+80.7 h, 95%CI 28.0, 133.3, *p* = 0.003), hemoglobin level < 10 mg/dL (+60.4 h, 95%CI 8.6, 112.3, *p* = 0.022), and serum albumin < 3.5 g/dL (+52.8 h, 95%CI 3.6, 102.0, *p* = 0.035) (Table 2). Sensitivity analysis results are presented in Supplementary Table S2.

## 4. Discussion

The identification of patients with acute heart failure who carry a higher risk of prolonged LOS is crucial for emergency physicians in arranging effective care and improving the ED throughput. In the full exploratory model study, four factors were independently associated with an increase in total LOS of patients with acute heart failure. These were high NYHA functional class, tachypnea, anemia, and hypoalbuminemia. After backward elimination, the reduced model showed elderly, defined as patients aged more than 65 years old, as another potential factor that could influence the LOS of patients with acute heart failure. All these factors are easily measured and can be promptly evaluated during an initial assessment in the ED.

In this study, patients with acute heart failure had similar clinical characteristics to those patients in developed countries, according to the figures reported by the United States (US) Acute Decompensated Heart Failure National Registry (ADHERE) in 2005 [28], the Euro Heart Failure Survey I (EHFS I) in 2003 [29], the Euro Heart Failure Survey II (EHFS II) in 2006 [30], and the Heart Institute of Japan Heart Failure II (HIJ-HF II). For instance, the mean age of our patients was 74 years (US ADHERE 72.5 years, EHFS I 71 years, EHFS II 69.7 years, HIJ-HF II 72 years). In terms of gender, our patients were predominantly male (62%), which was in concordant to the proportion of male patients in EHFS II (61%) and HIJ-HF II (71%). Less than half of the patients had reduced ejection fraction (23.1%), which is also consistent with proportions reported from studies in the US (46%) [28] and Japan (36%) [31]. Interestingly, the character of our study patients was quite different from the data reported by the Thai ADHERE study in 2010 [13]. Many factors contributed to the differences between the Thai ADHERE and our study: first, the Thai ADHERE included a large sample of patients from 18 hospitals with various levels of care. Second, the Thai ADHERE focused only on patients who were admitted to in-patient departments. Our study carried a smaller sample size, included all patients who visited the ED in one University hospital regardless of admission status, and was conducted almost a decade after the Thai ADHERE. Although the clinical characteristics of patients in our study and patients in developed countries had become more similar over the past decade, which might be the result of shifting toward a western lifestyle in developing countries [1], healthcare characteristics were not, especially in terms of admission rate. In our study, the overall admission rate, 34.8%, was significantly lower than that of the US ADHERE which was reported at 83.7% [5]. Limits in health care resources, the number of available beds, and the number of health care personnel in developing countries, including Thailand, may account substantially for this difference.

The NYHA functional classification is a well-known practical measure of severity in patients with heart failure [32,33,34,35,36]. The classification is based on the severity of symptoms and limitation of physical activities. It had consistently been proven as an independent predictor of mortality and poor clinical outcomes in either heart failure with reduced or preserved ejection fraction [2,3,4,5]. In our study, patients with higher NYHA functional class, especially for class III and IV, experienced longer LOS than patients with lower NYHA functional classes upon initial evaluation. This was consistent with a past study in elderly patients with decompensated heart failure [8]. Another recent study in Thailand reported that patients with NYHA class IV were eight times more likely to have longer LOS than patients with lower NYHA classes [14]. However, the median LOS in both studies (7.1 days and 7.5 days, respectively) were substantially longer than that of our study (2.3 days), as only hospitalized patients were included. Our study included all patients with acute heart failure who visited the ED, both admitted and not admitted. Moreover, both studies categorized LOS into two groups based on different cutoffs, LOS > 4 days in the first study and LOS > 7.5 days in the second study. Dichotomizing time variables reduces the statistical power of analysis, leads to loss of information, and could potentially bias the estimates [37,38]. Moreover, using different cutoffs may render the study results directly noncomparable.

Initial vital signs (i.e., respiratory rate, pulse rate, blood pressure, and oxygen saturation) are essential indicators for risk stratification of patients with acute heart failure [39]. Tachypnea (defined as respiratory rate > 24 breath per minute) was associated with an increase in the total LOS in this study. An increase in respiratory rate was clinical evidence of respiratory distress associated with mortality in many circumstances [40,41]. In patients with acute heart failure, it was rationalized that patients with higher respiratory rates were more likely to be intubated, admitted to an intensive care unit, or die in hospital [42]. Recently, an approach to categorize patients with heart failure based on the character of breathlessness was proposed [24]. In that study, the authors classified hospitalized heart failure patients into shortness of breath at rest (SOBAR) with a median respiratory rate of 24 (IQR 22, 29) and comfortable at rest but breathless on slight exertion (CARBOSE) with a median respiratory rate of 18 (IQR 17, 20). Interestingly, the authors found that there was no significant difference in the LOS between both groups, which was not in concordance with our findings. Differences in patients’ characteristics (in-patient and out-patient), classification criteria for breathlessness (other than respiratory rate), and healthcare provided might explain this discrepancy.

Anemia is commonly found in patients with acute heart failure. It leads to a decrease in oxygen delivery and aggravation of dyspneic symptoms, exercise intolerance, and impaired quality of life [43]. Several studies had reported its association with poor functional status [44], re-hospitalization [45], and mortality [46]. Patients with hemoglobin level < 10 g/dL were significantly associated with longer LOS in our study compared to patients with hemoglobin level > 10 g/dL. This finding was concordant to past studies [47,48,49]. Although the prevalence, characteristics, and impact of anemia might differ between patients with HFpEF and HFrEF [47], one population-based study had demonstrated the same direction of hemoglobin on mortality and the LOS [50].

In this study, hypoalbuminemia was identified as another significant factor associated with longer LOS in acute heart failure patients. Low serum albumin level is commonly found in patients with heart failure, both HFpEF and HFrEF [51,52]. Its presence was associated with worse heart failure outcomes and mortality [53]. Hypoalbuminemia reflects the presence of congestion [54], anemia [55], hyponatremia [55], inflammation [56], and malnutrition [57], all of which have a clinical impact on disease progression and mortality of patients with heart failure. For this reason, several nutritional assessment tools in hospitalized patients with heart failure had incorporated serum albumin in their patient evaluation [58,59]. In terms of LOS, one study in Japan reported that hypoalbuminemia was associated with prolonged LOS [21], which was in accordance with our results. However, only patients with HFpEF were included in that study, whereas our study included a broader spectrum of patients with heart failure.

Previously, several studies had identified other predictors of prolonged LOS in patients with heart failure such as gender [8,21], a number of comorbidities [9], severity of disease [9], and low systolic blood pressure (< 155 mmHg) on admission [21]. Almost all potential predictors included in our study showed statistical significance in univariable analysis, except for the male gender. Whether there was an association between gender and prolonged LOS in patients with acute heart failure is controversial. Our finding on gender was supported by a previous study in Puerto Ricans patients, which found no association between gender and LOS [60]. After adjusting for other predictors, only four factors were independently associated with an increase in LOS. Sensitivity analysis results were consistent with the primary analysis. Age ≥ 65 years was identified as another independent associating factor after the backward elimination of other non-significant predictors. The prolongation of hospital stay in the elderly could be the result of cognitive impairment, functional dependence, and higher comorbidity burden [61]. In addition, older patients with acute heart failure were at higher risk of complications, prolonged recovery, and mortality [62]. Many factors may contribute to the different findings in our study compared to previous reports, such as differences in the domain of patients, types of health care setting, statistical methods used, and predictors included in the multivariable analysis. Despite this, acute kidney injury (AKI) and worsening of renal function in patients with acute heart failure were reported to be associated with poor clinical outcome [27]. Thus, we did not include AKI and worsening of renal function as our candidate predictors as the objective was to identify predictors that can be readily identified upon the patient’s arrival to the ED without the need for further follow-up.

There were some limitations to our study. First, the patient data were retrospectively collected, which results in the exclusion of many patients with incomplete data on NT-proBNP, which could give rise to selection bias. It was identified from our data that patients who did not have data on NT-proBNP were relatively younger, had lower NYHA class, had higher systolic blood pressure, had higher oxygen saturation, and also had shorter LOS than patients who had the data on NT-proBNP. However, without NT-proBNP, the differentiation of acute heart failure from non-cardiac dyspnea might be less accurate. Moreover, the importance of using natriuretic peptides as inclusion criteria in clinical trials was recently proposed [63]. Thus, including patients without NT-proBNP results in statistical modeling might not be appropriate and may bias the results. The exclusion of these patients limits the generalization of our results to only patients with acute heart failure whose NT-proBNP level was more than 300 pg/mL. A further prospective study with a rigorous diagnostic protocol is required to confirm our findings before clinical implementation. Second, data on some clinically important parameters, which could also be potentially influencing factors of the LOS, were not available. These included the modified early warning score (MEWS) and comorbidity indices (e.g., Charlson Comorbidity index and Elixauser index). Nonetheless, as these parameters were not routinely evaluated or documented in our practice, identifying them as significant predictors might not be practical or applicable in our setting. In addition, our study aimed to examine the isolate influence of each potential predictor to the length of stay to identify the significant ones to be used together to identify patients at higher risk of prolonged length of stay in the ED. Third, as for routine practice, the provisional diagnosis of decompensated heart failure at the ED was left to physicians’ discretion, which might be subject to inter-rater reliability and inaccuracy. However, we validated the diagnosis of acute heart failure in all included patient records with the Framingham criteria and NT-proBNP, as suggested by the ESC guideline [19], to account for this issue. Fourth, patients with acute heart failure who presented with vague clinical symptoms were usually recorded with unspecific ICD-10 diagnoses (e.g., R06.00 dyspnea, unspecified or R07.9 chest pain, unspecified) in the hospital database and might not be included in our study. Fifth, due to limits in study size, we did not explore potential effect modification across different subgroups of patients, such as patients with different levels of left ventricular ejection fraction and hospitalization status. Finally, the findings were generated from a single center, which limits its generalizability.

## 5. Conclusions

This study identified four independent predictors of longer LOS in patients with acute heart failure from the main analysis. These were poor function class (NYHA class III/IV), tachypnea (RR > 24 per minute), anemia (Hb < 10 mg/dL), and hypoalbuminemia (serum albumin < 3.5 g/dL). Aged > 65 years was another potential factor that could influence the LOS identified from the sensitivity analysis. The presence of these factors could assist emergency physicians in prioritizing patients for hospital admissions when hospital beds are scarcely available.

## Figures and Tables

**Table 1:** Baseline clinical characteristics of the study patients (*n* = 207).

Characteristics	Mean ± SD or *n* (%)
Age (years)	74.2 ± 12.5
Gender	
Male	128 (62%)
Female	79 (38%)
Ethnicity	
Thai	204 (99%)
Non-Thai	3 (1%)
Underlying conditions	
Chronic kidney disease	99 (48%)
Ischemic heart disease	84 (41%)
Diabetic Mellitus	105 (51%)
Atrial fibrillation	84 (41%)
NYHA class	
0-I	36 (17%)
II	54 (26%)
III	92 (45%)
IV	25 (12%)
Vital signs	
BT (Celsius)	36.8 ± 0.6
SBP (mmHg)	152.4 ± 36.0
HR (/min)	90.6 ± 24.7
RR (/min)	25.5 ± 5.0
Oxygen saturation (%)	93.5 ± 5.4
Biochemistry	
NT-ProBNP (pg/mL)	14,240.1 ± 18,384.5
Serum Na (mmol/L)	136.8 ± 5.8
Hb (g/dL)	10.8 ± 2.1
Alb (g/dL)	3.0 ± 0.5

Abbreviations: SD, standard deviation; NYHA, New York Heart Association; BT, body temperature; SBP, systolic blood pressure; HR, heart rate; RR, respiratory rate; NT-ProBNP, N-terminal pro b-type natriuretic peptide; Na, sodium; Hb, hemoglobin; Alb, albumin.

**Table 2:** Estimated length of stay (LOS) and LOS difference between patients with and without predictor. Poisson mean of LOS and LO difference was estimated from univariable and multivariable Poisson regression.

Predictors	LOS (hours) Poisson Mean (SE)		Univariable Model		Multivariable Model	
	with Predictor	without Predictor	LOS Difference (hours) (95%CI)	*p*-Value	LOS Difference (hours) (95%CI)	*p*-Value
Demographic						
Age > 65 years	148.7 (1.0)	72.8 (1.2)	75.9 (72.9,79.0)	<0.001	40.5 (−7.3, 88.3)	0.097
Male	132.3 (1.3)	130.4 (1.0)	1.8 (−1.4, 5.1)	0.259	21.8 (−37.3, 80.9)	0.469
Comorbidities						
Chronic kidney disease	151.9 (1.2)	112.1 (1.0)	39.8 (36.6, 42.9)	<0.001	2.4 (−50.3, 55.2)	0.928
Ischemic heart disease	150.6 (1.3)	117.8 (1.0)	32.8 (29.5, 36.0)	<0.001	33.0 (−1.7, 81.7)	0.185
Diabetic mellitus	145.7 (1.2)	116.1 (1.1)	29.6 (26.5, 32.7)	<0.001	8.8 (−47.6, 65.2)	0.759
Atrial fibrillation	146.5 (1.3)	120.7 (1.0)	25.8 (22.6, 29.1)	<0.001	38.7 (−18.0, 95.4)	0.181
NYHA functional class						
III/IV	163.3 (1.2)	89.3 (1.0)	73.9 (70.9, 77.0)	<0.001	72.9 (23.9, 121.8)	0.004
Vital signs						
SBP (mmHg)						
<100	104.5 (3.9)	146.8 (1.3)	−42.4 (−50.4, −34.4)	<0.001	−48.0 (−130.7, 34.6)	0.255
100–140	Reference		Reference		Reference	
>140	121.4 (1.0)	146.8 (1.3)	−25.5 (−28.7, −22.2)	<0.001	−14.9 (−67.4, 37.6)	0.579
RR > 24 (/minute)	180.2 (1.4)	94.1 (0.9)	86.0 (82.7, 89.3)	<0.001	80.7 (28.0, 133.3)	0.003
Biochemistry						
NT-ProBNP ≥ 1800 pg/mL	138.1 (0.9)	95.5 (1.7)	42.6 (38.9, 46.3)	<0.001	16.1 (−1.4, 73.6)	0.583
Serum Na < 135 mmol/L	154.0 (1.4)	118.4 (0.9)	35.6 (32.2, 39.0)	<0.001	7.7 (−50.1, 65.4)	0.794
Hb < 10 g/dL	174.5 (1.4)	98.5 (0.9)	76.0 (72.7, 79.3)	<0.001	60.4 (8.6, 112.3)	0.022
Alb < 3.5 g/dL	146.7 (0.9)	59.5 (1.3)	87.2 (84.1, 90.3)	<0.001	52.8 (3.6, 102.0)	0.035

Abbreviations: LOS, length of hospital stay; SE, standard error; CI, confidence interval; NYHA, New York Heart Association; SBP, systolic blood pressure; RR, respiratory rate; NT-ProBNP, N-terminal pro b-type natriuretic peptide; Na, sodium; Hb, hemoglobin; Alb, albumin.

## Supplementary Materials

The following are available online at https://www.mdpi.com/1010-660X/56/9/434/s1, Table S1: Comparison of baseline clinical characteristics between patients who had data on NT-proBNP (*n* = 207) and patients who did not have data on NT-proBNP (*n* = 186), Table S2: Estimated LOS and LOS difference between patients with and without predictor from multivariable Poisson regression model together with the reduced model, and the sensitivity analysis model which includes baseline serum creatinine.
