# Supplementary material for: Clinical Predictors Influencing the Length of Stay in Emergency Department Patients Presenting with Acute Heart Failure
Source: Medicina (Kaunas). 2020 Aug 27;56(9):434. doi: 10.3390/medicina56090434 (PMC7558979; doi:10.3390/medicina56090434)
Supplement: Supplementary file 1 [file medicina-56-00434-s001.pdf]

Supplementary Table S1. Comparison of baseline clinical characteristics between patients who had data on NT-proBNP (n=207) and patients who did not have data on NT-proBNP (n=186).

Characteristics	Have data on NT-ProBNP (n=207)	No data on NT-ProBNP (n=186)	
	n (%)	n (%)	p value
Age (years, mean±SD)	74.2±12.5	71.3±11.6	0.017
Gender			
Male	79 (38%)	74 (40%)	0.757
Female	128 (62%)	112 (60%)	
Ethnicity			
Thai	204 (99%)	183 (100%)	
Non-Thai	3 (1%)	0(0)	
Underlying conditions			
Chronic kidney disease	99 (48%)	107 (58%)	0.056
Ischemic heart disease	84 (41%)	62 (33%)	0.145
Diabetic Mellitus	105 (51%)	104 (56%)	0.313
Atrial fibrillation	84 (41%)	53 (28%)	0.015
NYHA class			
0-I	36 (17%)	20 (11%)	0.008
II	54 (26%)	72 (39%)	
III	92 (45%)	83 (45%)	
IV	25 (12%)	11 (6%)	
Vital signs			
BT (Celsius, mean±SD)	36.8±0.6	36.8±0.6	0.775
SBP (mmHg, mean±SD)	152.4±36.0	160.5±36.0	0.027
HR (/min, mean±SD)	90.6±24.7	87.2±21.0	0.139
RR (/min, mean±SD)	25.5±5.0	24.7±4.8	0.123
Oxygen saturation (%, mean±SD)	93.5±5.4	94.8±6.4	0.027
Biochemistry			
NT-ProBNP (pg/mL, mean±SD)	14,240.1±18,384.5	-	
Serum Na (mmol/L, mean±SD)	136.8±5.8	136.9±6.0	0.781
Hb (g/dL, mean±SD)	10.8±2.1	10.8±2.5	0.827
Alb (g/dL, mean±SD)	3.0±0.5	3.0±0.5	0.951
Length of stay (hour, median (IQR))	54.6 (17.5, 149.3)	27.5 (6.8, 102.2)	<0.001

Abbreviations: NT-ProBNP, N-terminal pro b-type natriuretic peptide; SD, standard deviation; NYHA, New York Heart Association; BT, body temperature; SBP, systolic blood pressure; HR, heart rate; RR, respiratory rate; Na, sodium; Hb, hemoglobin; Alb, albumin; IQR, interquartile range.

Supplementary Table S2. Estimated LOS and LOS difference between patients with and without predictor from multivariable Poisson regression model together with the reduced model, and the sensitivity analysis model which includes baseline serum creatinine.

**Predictors**	**Multivariable model**		**Reduced model**		**Sensitivity analysis by including baseline serum creatinine**	
	**LOS difference (hours) (95%CI)**	**P-value**	**LOS difference (hours) (95%CI)**	**P-value**	**LOS difference (hours) (95%CI)**	**P-value**
Demographic						
Age >65 years	40.5 (-7.3, 88.3)	0.097	42.2 (1.19, 83.3)	0.044	39.1 (-8.38, 86.6)	0.106
Male	21.8 (-37.3, 80.9)	0.469	Not included		25.9 (-34.9, 86.7)	0.404
Comorbidities						
Chronic kidney disease	2.4 (-50.3, 55.2)	0.928	Not included		11.7 (-43.3, 66.7)	0.678
Ischemic heart disease	33.0 (-1.7, 81.7)	0.185	Not included		32.7 (-15.9, 81.4)	0.187
Diabetic mellitus	8.8 (-47.6, 65.2)	0.759	Not included		13.5 (-44.9, 71.9)	0.651
Atrial fibrillation	38.7 (-18.0, 95.4)	0.181	Not included		40.9 (-17.7, 99.3)	0.171
NYHA functional class						
III/IV	72.9 (23.9, 121.8)	0.004	72.3 (25.1, 119.5)	0.003	73.9 (24.7, 123.1)	0.003
Vital signs						
SBP (mmHg)						
<100	-48.0 (-130.7, 34.6)	0.255			-42.5 (-124.0, 39.0)	0.307
100-140	Reference		Not included		Reference	
>140	-14.9 (-67.4, 37.6)	0.579			-11.7 (-63.8, 40.3)	0.658
RR >24 (/minute)	80.7 (28.0, 133.3)	0.003	76.5 (23.8, 129.1)	0.004	80.0 (27.9, 132.2)	0.003
Biochemistry						
NT-ProBNP ≥1800 pg/mL	16.1 (-1.4, 73.6)	0.583	Not included		21.4 (-38.1, 80.8)	0.481
Serum Na <135 mmol/L	7.7 (-50.1, 65.4)	0.794	Not included		8.2 (-49.4, 65.8)	0.780
Hb <10 g/dL	60.4 (8.6, 112.3)	0.022	63.6 (11.3, 116.0)	0.017	65.1 (11.5, 118.7)	0.017
Alb <3.5 g/dL	52.8 (3.6, 102.0)	0.035	55.8 (5.9, 105.8)	0.028	55.9 (5.9, 106.0)	0.029

Abbreviations: LOS, length of hospital stay; SE, standard error; CI, confidence interval; NYHA, New York Heart Association; SBP, systolic blood pressure; RR, respiratory rate; NT-ProBNP, N-terminal pro b-type natriuretic peptide; Na, sodium; Hb, hemoglobin; Alb, albumin.
